# Cow Lying Behaviour and Bedding Quality Changes during Five Weeks on a Stand-Off Pad

**DOI:** 10.3390/ani9050257

**Published:** 2019-05-21

**Authors:** Cheryl O’Connor, Suzanne Dowling, Vanessa Cave, Jim Webster

**Affiliations:** AgResearch Ltd., Ruakura Research Centre, Private Bag 3123, Hamilton 3240, New Zealand; suzanne.dowling@agresearch.co.nz (S.D.); vanessa.cave@agresearch.co.nz (V.C.); jim.webster@agresearch.co.nz (J.W.)

**Keywords:** lying behavior, bedding quality, wood chips, dairy cattle, stand-off pads

## Abstract

**Simple Summary:**

New Zealand dairy farmers place cows in uncovered off-paddock facilities for a part (e.g., 18 h) of the day to reduce their impact on pasture during winter. Lying time is a key indicator of whether these hybrid pasture systems are meeting welfare requirements. While it is easy to measure lying time in a research setting using accelerometers, it is not yet common to measure it on farm, and more convenient indicators are needed. We investigated the lying behaviour of dairy cows as bedding quality deteriorated over a five-week period to determine what measures may be useful as farmer-friendly indicators of cow welfare. The daily lying time of the two groups of cows on bedding according to normal farm practice (NFP) declined over the five weeks and was significantly less than that of cows on fresh bedding during the last 10 days of the trial. The fresh woodchip bedding contained less than 65% moisture throughout the trial. By week 3 and week 4, the moisture content of the lying surface exceeded 75% for NFP Group 2 and Group1, respectively. A bedding moisture threshold of 75% assessed by a ‘gumboot score’ and cows lying rather than grazing when on pasture may be useful farmer-friendly indicators of cow welfare because of their relationship with reduced lying time.

**Abstract:**

Bedding quality and cow lying time were measured during five weeks in a normal farm practice (NFP) off-paddock system with no bedding refreshment. Two groups of 100 non-lactating dairy cows were compared to groups of 8 cows with fresh bedding (FB). The cows were on a woodchip pad for 18 h/d at a space allowance of 5.4 m^2^/cow, with 6 h/d on pasture for 5 weeks. Lying times were recorded continuously for 60 cows per group using accelerometers. Bedding moisture content was measured weekly. Data for each NFP group were analysed and compared with those of their respective FB group using repeated measures. The lying time declined over five weeks from 11.6 h/day during the first week to 5.6 h/day during the fifth week (SED = 0.3; F_1,25_ = 351.56; *p* < 0.001). The moisture content of the bedding increased over the five weeks and was significantly higher for both NFP groups (NFP Group 1: F_5,59_ = 8.33; *p* < 0.001; NFP Group 2: F_5,61_ = 5.54; *p* < 0.001) than those of the respective FB groups. The percentage of total time lying when in the paddock increased for the NFP groups, reaching 15% in the last week of the trial. During five weeks on a stand-off pad, bedding quality deteriorated, and cows lay down less, to such an extent that welfare was compromised.

## 1. Introduction

New Zealand has a pasture-based dairy system; however, the temporary use of off-paddock facilities is becoming more commonplace, especially to reduce environmental impacts. This is particularly done to protect the soil and consequently pasture production from treading damage such as pugging [1]. Having cows off pasture also reduces the risk of nitrogen leaching from urine and dung deposition [2]. Stand-off pads are uncovered facilities, predominantly used in winter when the cows are not lactating [3] and as a hybrid pasture system they have some time on pasture every day. 

Lying behavior is important for adult cattle and has been used as a measure of cow comfort in numerous studies. After a period in which cows were prevented from both lying down and feeding, cows delayed feeding to lie down [4]. In addition, they showed decreased plasma concentrations of growth hormone [5], elevated levels of plasma cortisol, and other indicators of stress after just a few hours when prevented from lying down [6,7]. Dairy cattle prefer, and spend more time, lying on soft, well-bedded [8,9,10,11] and dry [12,13] surfaces. Previous work has shown that housed cattle are motivated to lie down for approximately 12 h/d [14,15]. In New Zealand, cows on pasture spend approximately 10 h/d lying [16,17], and minimum lying times of 8 h/d are recommended to farmers in the dairy cattle code of welfare [18] and by industry advisors. However, there are constraints in the use of accelerometers and other research tools, such as video cameras, in pasture-based systems, making it hard for farmers to know if they are meeting this welfare requirement.

The key environmental variable which determines lying time and hence welfare and appropriate facility usage is bedding quality. Access to dry bedding has been shown to be important for dairy cows [13]. In uncovered facilities, bedding becomes wet, not just from rain but also when cows urinate and defecate and enter the lying area with dirty, wet hooves. Cows spent more time lying when they were stood off on a well-maintained wood-chip surface compared with a concrete surface [11]. Wet bedding has been shown to reduce the amount of time cows spend lying in free stalls [12].

In order to define good practice and to understand the welfare risks associated with the use of stand-off pads, we investigated the lying behaviour of dairy cows as bedding quality deteriorated over a five-week period. The aim of this study was to develop measures that farmers could use to determine if cows are receiving adequate rest and whether their welfare requirements are being met. To do this, we measured a suite of animal and bedding quality factors to determine their relationship to cow lying time.

## 2. Materials and Methods

Prior to the start of this study, ethical approval was obtained from the Ruakura Animal Ethics Committee (protocol no. 13566), as required by the New Zealand Animal Welfare Act 1999.

### 2.1. Animals

In this study, 216 healthy non-lactating dairy cows were divided into two groups of 100 cows, paired with two groups of 8 cows, balanced for breed, liveweight, age and expected calving date (Table 1). Cows were kept on an uncovered stand-off pad, with fresh woodchips (approx. 30 cm depth at the start of the trial) for 18 h, with 6 h on pasture (approximately from 9:00 a.m. to 3:00 p.m.) daily for 5 weeks. The duration of stand-off was chosen on the basis that common stand-off duration in New Zealand farms is 12–20 h/d [19]. Following normal farm practice, no feed was available on the pad, but water was available ad libitum. Each pad was 20 × 27 m, resulting in a stocking density of 5.4 m^2^/cow, again within the normal on-farm stocking rates for such a stand-off pad in New Zealand [19]. 

For each normal farm practice group (NFP) of 100 cows, a paired group of 8 cows each was kept at the same stocking density on 6 × 7.2 m pens with woodchips, which were refreshed three times a week, and represented the fresh bedding (FB) groups. Throughout the study, FB 1 measures were collected at the same time as NFP 1 measures and, likewise, for FB 2 and NFP 2 measures. Because of practical constraints and resource limitations, the incorporation of a proper control was not feasible. Instead, the smaller FB groups served as comparative groups.

Weather conditions, including air temperature (°C), relative humidity (%), rainfall (mm), solar radiation (watts/m^2^) and wind speed (m/s), were recorded throughout the trial at 10 min intervals using two portable weather stations (6163, Wireless vantage Pro2 Plus, Davis instruments, Harvard, CA, USA) located in an unsheltered area within 10 m of the stand-off pads.

### 2.2. Animal Measures

Lying times were recorded continuously for 60 cows per NFP group and five cows per FB group, using Onset Pendant G data loggers (Onset Computer Corp., Bourne, MA, USA) set to record *y*- and *x*-axes at 30 s intervals. The data loggers were placed in a durable fabric pouch and attached on the lateral side of the hind leg above the metatarsophalangeal point. The pouch was held in position by using Velcro patches, one sewn to the pouch, and the other glued to the leg of the cow. The pouch was further held in place by a strap around the leg of the cow. Data loggers were changed once, on day 15, for NFP Group 1 and both FB groups, and on day 17 for NFP Group 2.

Once a week, gait score for the same 60 cows was recorded on a 5-point scale (based on Thomsen [20]) when the cows were walking single-file down a lane on the way to their paddock for grazing. At the same time, each cow was given a dirtiness score [21] for their left side ranging from clean to very dirty on a 5-point scale (1 = no evidence of manure contamination, 2 = some splashing, 3 = some clumps, 4 = moderate clumps and 5 = many manure clumps) for each of five areas: flank, ventral abdomen, udder, upper and lower rear leg. One person recorded the gait score, and one person the dirtiness score throughout the trial.

### 2.3. Bedding Measures

Bedding measures from 10 stratified 25 × 40 cm quadrat samples across the gate, middle, edge and trough areas of each pad were taken weekly. Similarly, five samples were taken from the FB 1 and two pens at the same time. Samples of approximately 100 g were taken, weighed and dried at 70 °C for two days and then reweighed to calculate the percentage of dry matter (reported as moisture content).

A visual score on a scale of 0–3, ranging from 100% wood chips to greater than 75% mud/manure was taken along with a “Gumboot score” on a scale of 0–3 to describe the degree of muddiness of the lying surface:

0—Clean, dry appearance woodchips—not distorted by standing on and nothing sticking to boots
1—Mixture mud/woodchips, some mud sticking to the bottom of boots, spreading slightly on standing
2—Surface soft/muddy, spreads on standing, and boots are left muddy
3—Surface may be runny or deep mud, i.e., it covers feet and stays on boots or it is difficult to lift the feet up out of “mud”

### 2.4. Statistical Analysis

Animal measures and bedding quality data for each NFP group were analysed separately with those of their appropriate FB group (i.e., groups were measured on the same days). Unless otherwise stated, all analyses were conducted using GenStat18 version 19 [22].

The data from the data loggers were downloaded using the HOBOware Pro software (Onset Computer Corp., Pocasset, MA, USA) and converted to daily summaries of lying behaviour using the SAS software [23] code designed for this purpose, on the basis of the work of Ledgerwood [24], correcting for single standing and lying events. Daily lying time data were analysed using repeated measures, formulated as a mixed model and fitted using Residual Maximum Likelihood. The fixed effects were Group, Time and the Group by Time interaction. The correlation between measurements taken on an individual cow over time was modelled using a power model of order 1. Fisher’s protected least significant difference at the 5% level was used to compare the NFP and FB group means over time (Time). In addition, daily lying times at week 1 and 5 of the study were compared for the NFP groups using one-way ANOVA blocked by group.

The bedding quality data were analysed using repeated measures, formulated as a mixed model. The correlation between bedding measurements made on an individual quadrat over time was modelled using a power model of order 1. The fixed effects of Group (NFP, FB), Area (gate, middle, edge and trough), Time and their interactions were tested, with the model selected by sequentially dropping statistically non-significant terms (tested at the 5% significance level). Fisher’s protected least significant difference at the 5% level was used to compare the NFP and FB group means. 

The weekly data for an average dirtiness score, i.e., total dirtiness/5, were analysed using repeated measures, formulated as a mixed model with effects for Group, Time and Group by Time interaction. The correlation between the measurements taken for an individual cow over time was modelled using a power model of order 1. Fisher’s protected least significant difference at the 5% level was used to compare the NFP and FB group means over time (Time). 

## 3. Results

**Figure 1:**
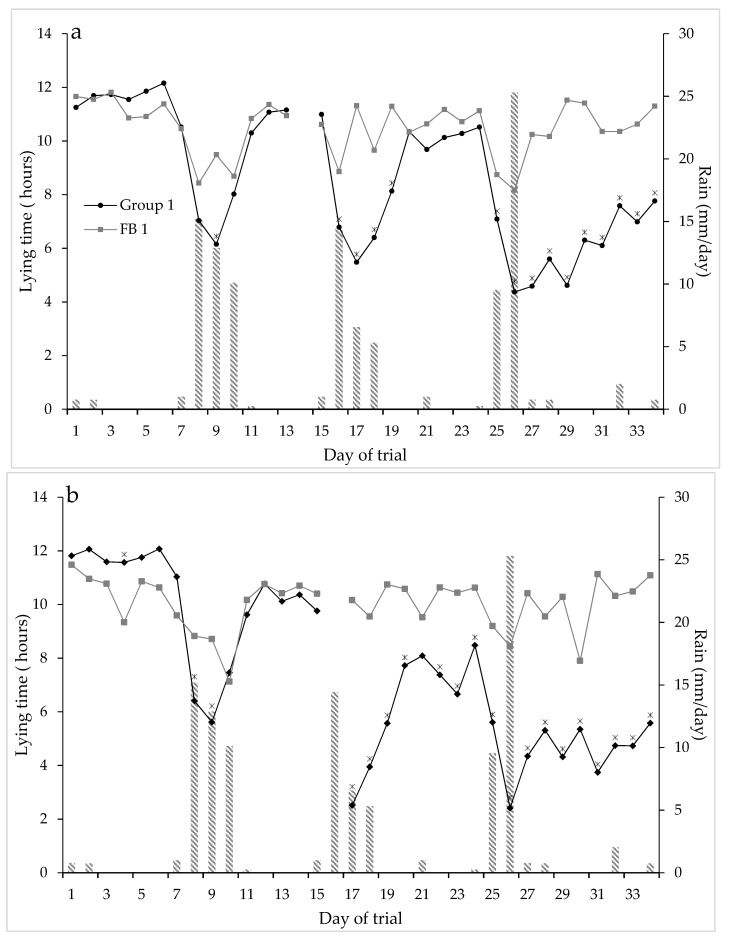
Total daily lying time (hours) over the 34 days of the trial for NFP Group 1 and FB 1 (**a**) and NFP Group 2 and FB 2 (**b**) (* indicates significance, *p* < 0.05, between the groups at each time point) over the five weeks of the trial. (Data logger change over days were excluded from the data). Rainfall (mm/day) during the trial period is also shown as bars.

The average daily temperature over the trial was 9.4 °C (the min temperature recorded was −2.1 °C, and the maximum temperature was 18.6 °C). There was some rain on 21 of the 35 days of the trial; however, only on 8 days it was over 5 mm (Figure 1). These rainfall events coincided with the decreases in lying time around days 9, 17 and 26 of the trial (Figure 1). 

The daily lying time for the NFP groups declined over the five weeks from 11.6 h/d during the first week to 5.6 h/d during the fifth week (SED = 0.326; F_1,25_ = 351.56; *p* < 0.001; Figure 1). The FB groups maintained a reasonably consistent daily lying time of 10.5 h/d throughout the five weeks (Figure 1). The daily lying times of the NFP1 (test of Time.Group: F_32,223_ = 8.27; *p* < 0.001) and NFP 2 (test of Time.Group: F_30,238_ = 10.18; *p* < 0.001) groups were statistically significantly lower than those of the FB groups for the last 10 days (i.e., from day 20) and after a fortnight on the pad (i.e., from day 17), respectively. 

**Figure 2:**
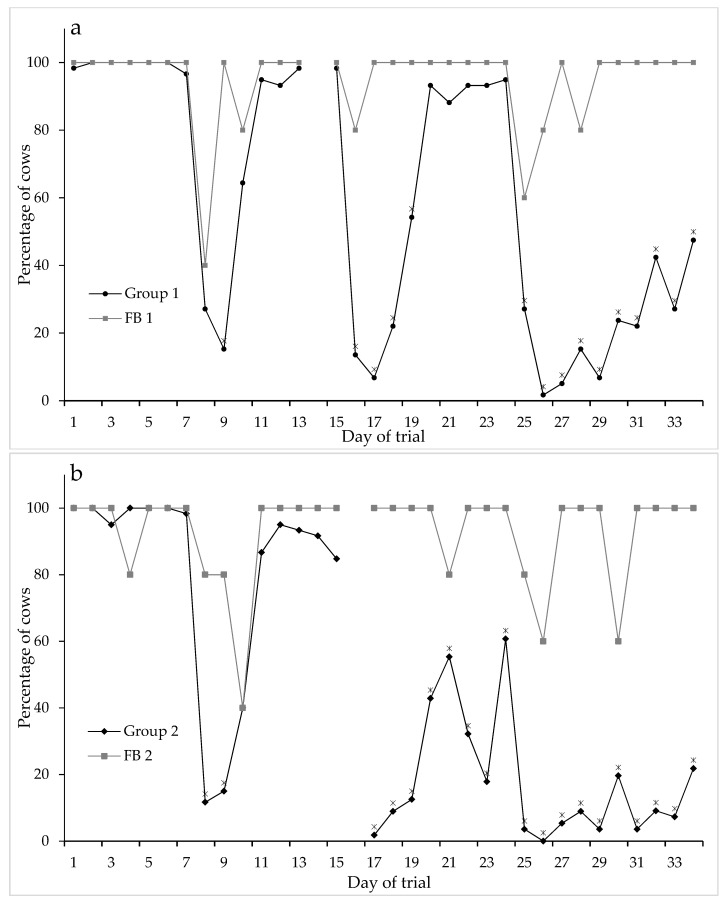
Percentage of cows that achieved at least 8 h lying per day, for NFP Group 1 and FB 1 (**a**) and NFP Group 2 and FB 2 (**b**) (* indicates significance, *p* < 0.05, between the groups at each time point) over the five weeks of the trial. (Data logger change over days were excluded from the data).

The percentage of cows that achieved 8 h lying decreased over time and was significantly different from the corresponding percentage in the FB groups on the same days considering total daily lying time, i.e., for the last 10 days for the NFP Group 1 (Figure 2: test of Time.Group: F_32,388_ = 5.92; *p* < 0.001) and after 2 weeks on the pad (i.e., from Day 17) for the NFP Group 2 (Figure 2: test of Time.Group: F_30,289_ = 6.42; *p* < 0.001).

**Figure 3:**
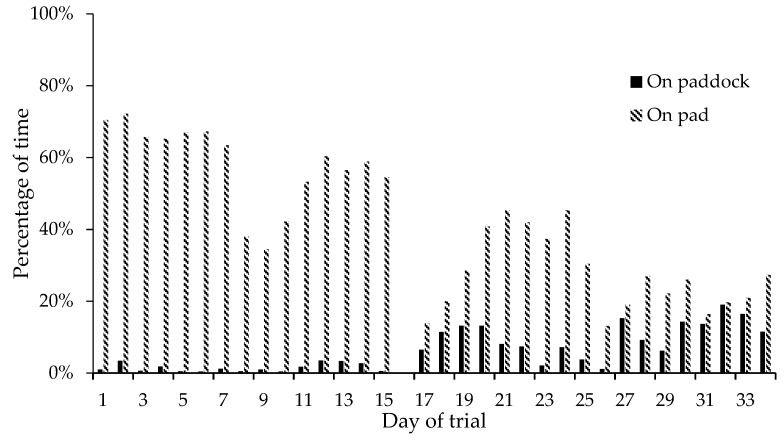
Mean percentage of time spent lying when on the pad or paddock for NFP Group 2. (Data logger change over (day 16) was excluded from the data).

The decline in daily lying time was mainly driven by a decline in lying time on the pad. For NFP Group 2 (and to a lesser extent NFP Group 1), the time spent lying in the paddock increased after approximately two weeks, however, this did not compensate for the reduction of time spent lying on the pad. The percentage of time spent lying on the pad declined from over 65% in week 1 to less than 30% in week 5 (Figure 3). At the same time, the percentage of time lying in the paddock increased, reaching 15% of time in the paddock in the last week of the trial (Figure 3). The effect was most marked during the first three hours on pasture, when less than 10% of cows were lying, at the start of the trial, and throughout the five weeks for the FB groups. However, in the last week of the trial, more than 40% of the cows were lying down during the first three hours in the paddock. 

**Figure 4:**
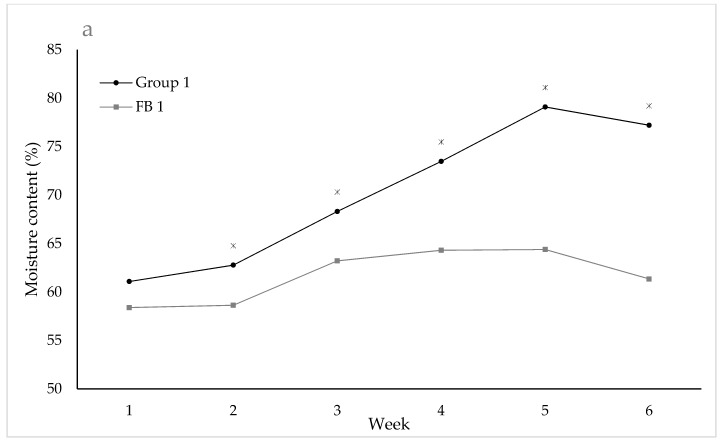

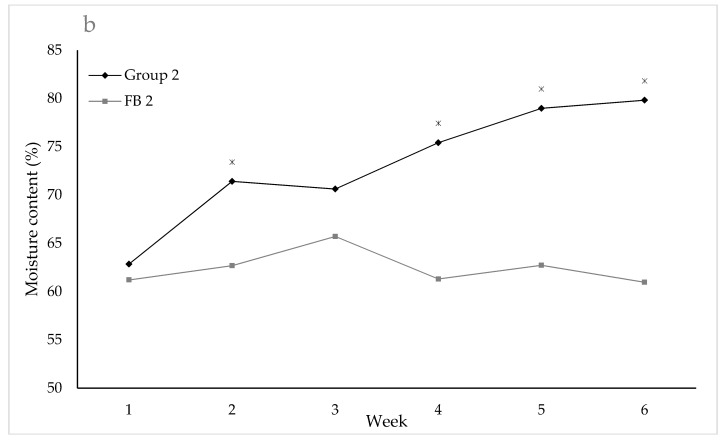
Mean moisture content (%) of the bedding for NFP Group 1 and FB 1 (**a**) and NFP Group 2 and FB 2 (**b**) (* indicates significance, *p* < 0.05, between the groups at each time point) over the five weeks of the trial.

In NFP Group 1, the moisture content of the bedding differed significantly between areas of the pad (test of Area: F_3,27_ = 6.85; *p* = 0.001: Trough 63.5%; Middle 65.1%; Gate 67.1%; Edge 68.4%; LSD (5%) ≈ 2.4). The moisture content of the bedding increased over the five weeks and was significantly higher for both NFP groups (test of Time.Group: NFP Group 1: F_5,59_ = 8.33; *p* < 0.001; NFP Group 2 F_5,61_ = 5.54; *p* < 0.001) than for the FB groups from week 1 (Figure 4; except for week 2, NFP Group 2). The woodchip bedding in the FB groups contained less than 65% of moisture throughout the trial. By week 3 and week 4, the moisture content of the lying surface was over 75% for NFP Group 2 and NFP Group 1 respectively (Figure 4). 

The mean visual score never exceeded 0.8 for the FB groups of cows on the refreshed woodchips. In comparison, from week 1 (NFP Group 2) and week 2 (NFP Group 1), the mean visual (test of Time.Group: NFP Group 1: F_5,62_ = 4.47; *p* = 0.002; NFP Group 2 F_5,57_ = 3.51; *p* = 0.008) and the gumboot scores (test of Time.Group: NFP Group 1: F_5,60_ = 6.18; *p* < 0.001; NFP Group 2 F_5,60_ = 3.94; *p* = 0.004), were significantly increased (Table 2). Although the NFP groups had similar mean scores, the texture of the bedding was more sticky “mud” for NFP Group 1 and deep and runny for NFP Group 2. 

**Figure 5:**
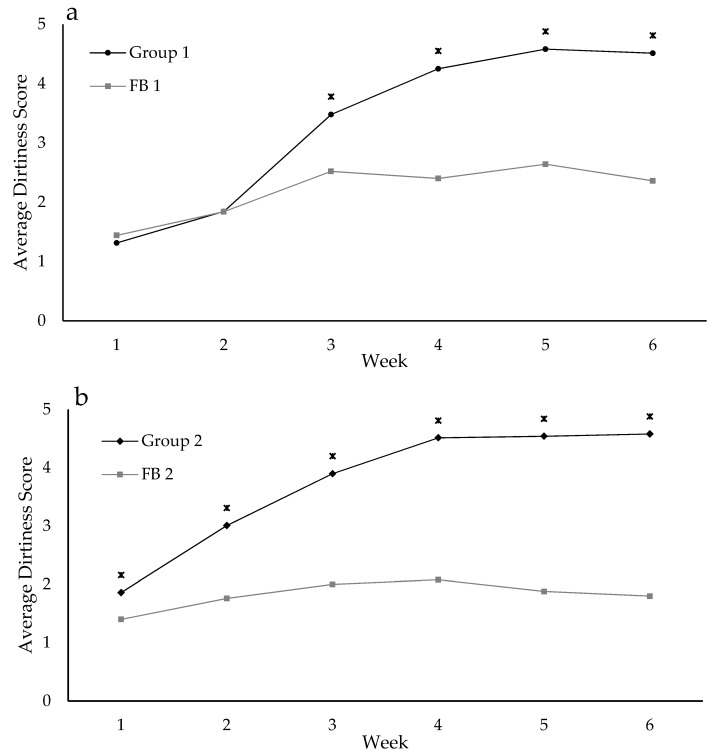
Average dirtiness score for NFP Group 1 and FB 1 (**a**) and NFP Group 2 and FB 2 (**b**) (* indicates significance, *p* < 0.05, between the groups at each time point) over the five weeks of the trial.

Cow dirtiness score increased over the trial until reaching a maximum at week 3 for NFP Group 2 and week 4 for NFP Group 1 (Figure 5). The average dirtiness scores were significantly higher than those of the FB groups throughout the trial for NFP Group 2, and from week 2 for NFP Group 1 (Figure 5). There was no effect of time on stand-off on the gait score or on the order the cows left the pad for the paddock.

## 4. Discussion

The daily lying time of non-lactating dairy cows declined during a five-week period in a typical New Zealand winter stand-off pad/pasture hybrid system. The management of an uncovered woodchip pad in this manner (18 h/day) produced a surface that cows were reluctant to lie on within three weeks and resulted in cows being severely deprived of lying. Lying times of less than 6 h per day have been shown to cause stress-induced perturbations of the hypothalamic–pituitary–adrenal axis [6]. Lower lymphocyte and basophil counts have also been measured in cows in muddy conditions [25] or exposed to wet winter conditions in New Zealand [26]. Reductions in circulating lymphocyte counts are sometimes interpreted as a sign of immunosuppression [27]. This, combined with poorer hygiene in muddy conditions, as indicated in this study by increasing dirtiness, could present an increased risk of mastitis. Although wet underfoot conditions are known to soften hooves and therefore increase the risk of lameness [28,29,30], we found no effect of time on the stand-off pad on the gait score. 

For comparison, the small group of cows on a pad with refreshed woodchips achieved lying times of 10.5 h/d throughout the five weeks of the trial. This lying time is similar to those other studies have measured with shorter exposures to woodchip pads [11] and for cows on pasture, which also spend approximately 10 h lying per day [13,16]. This illustrates the importance of frequent refreshing of the bedding to achieve adequate lying times; however, refreshing bedding is not common practice for an uncovered outdoor stand-off pad. This study has therefore highlighted a management option for farmers that could extend the period of use for a stand-off pad whilst meeting cow resting needs. 

There was a significant effect of rain and time of rainfall on the average daily lying time. The lying time decreased 13 min per day per mm of rainfall. There was also a cumulative effect of the rainfall on bedding moisture content, particularly from rainfall events in the preceding 48 h, which had a resulting effect on cow lying times. 

Many studies have shown that cows prefer a dry surface to lie on [12,13], and our results extend these findings by confirming that a surface with a moisture level above 75% is too wet to be a comfortable lying surface. Surface moisture is affected by the number of cows, the length of time cows are on the pad, the space the cows have and rainfall. Cattle generally have a high resistance to cold weather but, if they have to lie down on wet, muddy surfaces in cold conditions, this will increase heat loss due to conduction to the colder surface and may lead to issues with thermoregulation and cold stress [31,32,33]. The decision by the cow not to lie down when the bedding moisture content exceeds 75% may be partly due to increased heat loss. Recent research suggests that wet surfaces influence lying times and the quality of rest. Cows on wet woodchips spent less time lying in a lateral position and with their heads supported, indicating reduced cow comfort and quality of rest on this surface [21]. Severely reduced lying times on muddy surfaces compared to dry surfaces have also been reported [25,31,32]. In addition, evidence from New Zealand suggests that it is the wetness of the muddy surface, rather than the contamination with manure, that is aversive to cattle [21]. The bedding scores developed and used during the trial provided a good indicator of the bedding condition. A combination of the visual score and gumboot score appeared to provide a good indication of bedding quality, showing deterioration of the surface in line with the observed increased moisture content of the bedding.

As in other winter studies [31,32], cows got dirtier as bedding moisture content increased. Dirtiness is a multi-faceted welfare concern, with both health and production impacts. Poor udder hygiene is associated with higher somatic cell scores [34] and uterine and intra-mammary environmental pathogens [35], which predispose dairy cattle to endometritis [36,37] and mastitis. While mastitis is primarily a concern for lactating cows, infections during the dry period may persist into lactation, leading to clinical cases. 

As daily lying time on the pad decreased, cows lay down more when in the paddock, a consistent response even with shorter periods of stand-off than those used in this study [11,32], Schütz [38] determined that cows that are not able to lie down for a sufficient amount of time during the stand-off period will try to compensate by lying down more when they are at pasture, a time when they ideally should be grazing. Although the cows in this study did not have the high energy requirements associated with lactation, they were in late pregnancy, and it is unclear whether less than 6 h of grazing per day is sufficient to meet their daily energy requirements. From our findings, it appears that, when tired, cows were experiencing a trade-off between grazing and resting during the time on pasture and chose to graze for an hour or two to satisfy immediate hunger needs and then lie down to meet their resting needs. Others have found that, after a deprivation period of both eating and lying, cattle choose lying over eating [4,39]. Another potential on-farm indicator of whether cows are achieving adequate lying times, therefore, is the behaviour of the cows when they return to pasture. This study found that the lying pattern of the herd changed when the stand-off pad became too wet to lie comfortably, with more cows lying down sooner, and for longer, when on the pasture.

In conclusion, during five weeks in a typical New Zealand stand-off system, bedding surfaces became unsuitable for lying, so that cows failed to meet the recommended daily lying times. We identified several indicators that could assist farmers to manage the quality of the bedding in such a system. A threshold of 75% bedding moisture correlated with the ability of the cows to achieve an adequate lying time, and this could be readily assessed by a combination of the visual and gumboot scores. Cow lying behaviour assessed in the paddock was an additional indicator of cow welfare due to its relationship with reduced lying times on the stand-off pad. 

## Figures and Tables

**Table 1:** Number for breed type and mean ± SD liveweight, age and calving date for the trial groups. NFP: normal farm practice, FB: fresh bedding.

	NFP 1 (*n =* 100)	FB 1 (*n =* 8)	NFP 2 (*n =* 100)	FB 2 (*n =* 8)
Breed (Number Holstein–Friesian: Friesian × Jersey: Other)	53:44:3	5:3:0	61:35:4	4:3:1
Average liveweight (kg)	449.6 ± 51.7	490.6 ± 61.8	463.1 ± 49.0	482.4 ± 52.8
Average age (years)	5.0 ± 1.9	6.5 ± 2.7	5.9 ± 2.3	6.0 ± 2.6
Average calving date (weeks after end of trial)	7.3 ± 2.9	7.0 ± 2.9	7.6 ± 3.0	6.8 ± 2.3

**Table 2:** Mean visual and gumboot scores (measured on a 0–3 scale) over the five weeks of the trial. Fisher’s Least Significant Difference at the 5% level (LSD (5%)) was used to compare the means of a group over time.

Week	Mean Visual Score				Mean Gumboot Score			
	NFP 1	FB 1	NFP 2	FB 2	NFP 1	FB 1	NFP 2	FB 2
0	0.4	0.2	0.5	0.4	0.1	0.02	0.2	0.4
1	0.5	0.2	1.9 *	0.0	0.4	0.02	1.4 *	0.0
2	1.6 *	0.4	1.8 *	0.4	1.1 *	0.2	1.4 *	0.2
3	2.3 *	0.8	2.3 *	0.2	1.5	0.8	2.0 *	0.2
4	2.7 *	0.2	2.7 *	0.0	2.4 *	0.2	2.1 *	0.0
5	2.7 *	0.0	2.6 *	0.4	2.5 *	0.02	2.2 *	0.2
LSD (5%)	0.86		0.98		0.73		0.84	

* denotes weeks where significant difference between NFP and FB groups were measured.
